# Effects of Dietary Supplementation with Bilberry Extract on Growth Performance, Immune Function, Antioxidant Capacity, and Meat Quality of Yellow-Feathered Chickens

**DOI:** 10.3390/ani11071989

**Published:** 2021-07-02

**Authors:** Yibing Wang, Xinyan Ma, Jinling Ye, Sheng Zhang, Zhilong Chen, Shouqun Jiang

**Affiliations:** State Key Laboratory of Livestock and Poultry Breeding, Key Laboratory of Animal Nutrition and Feed Science in South China, Ministry of Agriculture and Rural Affairs, Guangdong Provincial Key Laboratory of Animal Breeding and Nutrition, Institute of Animal Science, Guangdong Academy of Agricultural Sciences, Guangzhou 510640, China; wangyibing77@163.com (Y.W.); dk051maxy@163.com (X.M.); YeJL2014@163.com (J.Y.); zhangszin@163.com (S.Z.); folder737497@163.com (Z.C.)

**Keywords:** antioxidant capacity, bilberry extract, immune function, meat quality, yellow-feathered chickens

## Abstract

**Simple Summary:**

Various plant extracts are used as functional nutritional factors to keep the health and improve the performance of animals. This research investigated the effects of bilberry extract (effective ingredient: anthocyanin) on growth performance, meat quality, antioxidant status, and immune function of yellow-feathered chickens. Results showed that dietary supplementation with bilberry extract enhanced relative weight of the bursa of Fabricius and broadly increased activities of antioxidant enzymes of chickens; indices of meat quality were improved without impact on growth performance. The finding indicated that bilberry extract might be considered as a new additive to improve the health and meat quality of yellow-feathered chickens.

**Abstract:**

The experiment was conducted to investigate the effects of bilberry extract on growth performance, meat quality, antioxidant status, and immune function of yellow-feathered chickens. A total of 360 female hatchling Lingnan chickens were randomly allocated to three treatments with 6 replicates of 20 chickens per replicate. Birds were fed a basal diet supplemented with 0 (the control group), 100 (B100), and 400 (B400) mg/kg of bilberry extract for 63 d. Compared with the controls, (1) dietary supplementation with bilberry extract did not affect the growth performance of chickens from 1 to 63 d. (2) At 21 d, the relative weight of the bursa of Fabricius was increased (*p* < 0.05) by dietary supplementation with 400 mg/kg bilberry extract. Bilberry extract decreased the concentrations of IgY and IgM in blood plasma of 63-d chickens (*p* < 0.05). (3) For 21-d chickens, dietary supplementation with 400 mg/kg bilberry extract increased (*p* < 0.05) the activity of GSH-Px in blood plasma and jejunal mucosa (*p* < 0.05). Supplementation with 100 mg/kg bilberry extract increased (*p* < 0.05) the activities of T-SOD in jejunal mucosa and GSH-Px in the liver and decreased (*p* < 0.05) the MDA concentration in the liver. For chickens at the age of 63 d, both levels of bilberry extract increased activity of T-SOD in blood plasma (*p* < 0.05) and reduced MDA concentration in the jejunum (*p* < 0.05). (4) Supplementation with bilberry extract in the diet decreased the MDA concentration (B100) in muscle of 63-d chickens at 45 min postmortem and increased (*p* < 0.05) the activity of T-SOD (B400) at 4 d postmortem. (5) In breast muscle at 63 d, birds supplemented with bilberry extract (B400) had increased pH and drip loss while drip loss was reduced in the B100 treatment (*p* < 0.05); treatments did not affect inosinic acid or intramuscular fat contents. In conclusion, dietary supplementation of yellow-feathered chickens with bilberry extract enhanced the relative weight of the bursa of Fabricius, and broadly increased activities of antioxidant enzymes; indices of meat quality were improved without impact on growth performance. Considering the results in the current research, 100 mg/kg bilberry extract was recommended when supplemented in chickens.

## 1. Introduction

In recent years, plant extracts have been widely used as functional nutritional factors to maintain health and improve the performance of animals. As the result of antibacterial, antiviral, and antioxidant properties of the bioactive components [1], plant extracts were proved to improve the productive performance [2], the intestinal environment [3], and enhance the immune function of animals [4]. Furthermore, plant extracts were able to slow down the extent of oxidation of meat samples thus prolonging the duration over which chicken meat was stored [5].

Anthocyanins, as the major bioactive component of bilberry extract, are water-soluble flavonoids [6], shown to possess a range of pharmacological effects, such as antioxidant, anti-inflammatory [7], anti-tumor, antibacterial, anti-mutagenic, and neuroprotective properties [8,9,10]. A study in male Zucker fatty rats showed that supplementation with 4% tart cherry anthocyanins in the diet for 8 weeks reduced mRNA levels of pro-inflammatory markers including tumor necrosis factor-alpha (*TNF-α*), interleukin-beta (*IL-β*), *IL-6*, and inducible nitric oxide synthase (*iNOS*) in adipose tissue, indicating that anthocyanins regulated immune function and reduced inflammation [11]. Cranberry juice (high in anthocyanins) increased the antioxidant capacity of the liver, heart, and kidney of hamsters [12]. Raspberry extract (rich in anthocyanins) reduced the ROS levels, increased GSH content, and ameliorated H_2_O_2_-induced oxidative stress in HepG2 cells via the Keap1/Nrf2 pathway [13]. As far as we known, bilberry anthocyanin has been mainly used in human health care or in studies in rats, but there has been little research about its application in livestock or poultry.

As an important strain of chickens, yellow-feathered chickens are preferred by customers by the great meat quality and strong meat flavor. Nowadays, the production of yellow-feathered chickens is approximately four billion annually, almost the same as white-feathered chickens. Among them, after improvement, fast-growing strains reach market weight in 63 days. According to the above research, a hypothesis that bilberry anthocyanins play antioxidant and immune-regulated roles, thus improving the meat quality and promoting the healthy production of chickens, was put forward. This experiment was conducted to investigate the effects of dietary supplementation with bilberry extract on growth performance, antioxidant capacity, immune function, and meat quality of yellow-feathered chickens, thus providing a basis for its potential application in broiler production.

## 2. Material and Methods

### 2.1. Chicken Husbandry

The experimental protocol was approved by the Animal Care Committee of the Institute of Animal Science, Guangdong Academy of Agriculture Science, Guangzhou, P. R. China, with the approval number of GAASIAS-2019-007. A total of 360 one-day-old, yellow-feathered chickens (Lingnan, female) with similar initial body weights (42 ± 0.17 g) were randomly allotted to 3 treatments, each with 6 replicates, and 20 birds per replicate, in a completely randomized design. Feed and water were available ad libitum until d 63, the typical age of marketing. Daylight was eliminated and replaced with 18-h lighting from incandescent bulbs. The temperature of the room was maintained at 32 to 34 °C for the first 3 days and then reduced by 2 to 3 °C per week to a final temperature of 26 °C.

### 2.2. Experimental Diets

The basal corn and soybean-meal diet was formulated according to the recommendation of Nutrient Requirements of Yellow Chicken [14]. Details of ingredient composition and calculated nutrient contents of the basal diets for chickens are provided in Table 1. Dietary treatments consisted of the basal diet supplemented with 0 (CON), 100 (B100), or 400 (B400) mg/kg bilberry extract, respectively. Bilberry extract (61% anthocyanins) was purchased from Tianjin Jianfeng Natural Products Co., Ltd. (Tianjin, China), which was extracted from bilberry using ethanol.

### 2.3. Measurement of Growth Performance

Feed intake was recorded daily on a per replicate basis. Birds were weighed per replicate at the beginning (d 1) and the end of each growth phase (d 21, d 42, and d 63). Mortality was checked daily, and dead birds were recorded and weighed to adjust estimates of gain, intake, and feed conversion ratio, as appropriate. The final bodyweight (BW), average daily feed intake (ADFI), average daily gain (ADG), and feed/gain ratio (F/G) were calculated.

### 2.4. Analysis of Carcass Traits

At the end of the starter phase (d 21), 2 birds close to average BW in each replicate were chosen, deprived of feed overnight, and weighed immediately prior to slaughter. The birds were electrically stunned (head only) at 150 V for 5 s (DMJ, Ningguang Machinery Co., Ltd., Nanjing, China) and exsanguinated. The spleen, thymus, and bursa of Fabricius were dissected, blotted, and weighed. Relative weight of immune organs = the immune organ weight/live weight × 100%.

### 2.5. Sample Collection

To study the antioxidant effect of blueberry extract in most cases of increased oxidative stress, the time intervals were characterized by the presence of potential oxidative damage as a result of feed changes, such as temporary dysfunction of the intestinal barrier (21 d) or as a result of organism aging and related intensification of inflammatory processes (63 d).

For 21-d chickens chosen in Section 2.4., the birds were bled from the right brachial vein into evacuated EDTA-K2 tubes before slaughtering. Blood samples were centrifuged at 1000× *g* for 15 min at 4 °C to obtain blood plasma, which was then stored at −80 °C for biochemical determinations. A subsample of liver was frozen (−80 °C). The jejunum was collected for the following research, as the small intestine is not only the main organ of nutrient absorption, but also the component of the intestinal barrier. Mid-jejunal segments were carefully dissected, opened lengthwise, and rinsed with sterile saline. The mucosa was collected immediately by gentle scraping and frozen in liquid nitrogen.

At the end of the whole growth phase (d 63), 2 birds close to average BW in each replicate were chosen and deprived of feed overnight. Blood plasma was collected as described above. Jejunal samples from 63-d chickens were collected and treated as previously described. Two pieces of breast muscle were collected. One piece was used for the determination of meat quality. The other piece was divided into two parts, with one part frozen in liquid nitrogen at 45 min post-mortem, while the other was frozen in liquid nitrogen after storage at 4 °C for 4 d.

### 2.6. Determination of Immunoglobulin Concentration

Samples of jejunal mucosa were homogenized with ice-cold physiologic saline (1:10, *v*/*v*) and centrifuged at 2000× *g* for 10 min to obtain clarified homogenates.

The content of IgA, IgY, and IgM in blood plasma and jejunal extracts of chickens at d 21 and d 63 were determined by ELISA kits (Nanjing Jiancheng Institute of Bioengineering, Nanjing, China) and a spectrophotometer (Biomate 5, Thermo Electron Corporation, Rochester, NY, USA).

### 2.7. Determination of Biochemical Variables

Samples of muscle were homogenized with ice-cold physiologic saline (1:10, *v*/*v*) and centrifuged at 2000× *g* for 10 min to clarify the homogenates.

Colorimetric kits (Nanjing Jiancheng Institute of Bioengineering) were used to assay the activities of total superoxide dismutase (T-SOD), glutathione peroxidase (GSH-Px), diamine oxidase (DAO), inducible NO synthase (iNOS) and the content of malondialdehyde (MDA) and NO in blood plasma, the activities of GSH-Px, T-SOD, catalase (CAT), total antioxidant capacity (T-AOC), and the content of MDA in liver and jejunum. Moreover, the activities of GSH-Px, T-SOD, and the content of MDA in muscle 45 min and 4 d postmortem were assayed.

### 2.8. Determination of Flavor Components in Muscle

Muscle tissue was cut into small pieces, lyophilized, and powdered. The intramuscular fat (IMF) was determined by Soxhlet extraction (FOSS 2055, Hilleroed, Denmark). The results are expressed as the content of total fat as a percentage of the lyophilized powder. The content of inosinic acid was entrusted to Guangzhou KingMed Diagnostics Group Co., Ltd. (Guangzhou, China) for determination using high-performance liquid chromatography (HPLC). In brief, the muscle sample (2 g) was smashed and homogenized. The sample was extracted with 5% perchloric acid solution and then reacted with 0.5 mol/L sodium hydroxide solution to form chemically stable sodium inosine. The content of inosinic acid was determined by HPLC (1260, Agilent Technologies Co., Ltd. (Santa Clara, CA, USA) and quantified by an external standard method.

### 2.9. Determination of Meat Quality

The piece of breast muscle for determination of meat quality was kept at 4 °C. Objective indices related to meat quality of breast muscle, including shear force, drip loss, instrumental (L*, a*, and b* value) color (45 min and 24 h post-mortem), and pH (45 min, 24 h, and 4 d post-mortem) were determined, as described by Wang et al. (2019).

### 2.10. Statistical Analysis

The effects of treatment were examined by one-way analysis of variance (ANOVA) in SPSS 20.0 for Windows. When treatment effects were significant (*p* < 0.05), Duncan’s multiple range tests were used to compare pairs of means. Tabulated results are shown as means with SEM derived from the ANOVA error mean square.

## 3. Results

### 3.1. Growth Performance

As shown in Table 2, there was no effect (*p* > 0.05) of bilberry extract on BW, ADG, ADFI, F/G, and survival rate of birds from 1 to 63 days of age.

### 3.2. Relative Weight of Immune Organs

For 21 d chickens (Table 3), compared with the control group, the relative weight of bursa of Fabricius was significantly increased (*p* < 0.05) by supplementation with 400 mg/kg bilberry extract in the diet. There was no effect on the relative weight of the liver, spleen, and thymus (*p* > 0.05).

### 3.3. Content of Immunoglobulin in Blood Plasma and Jejunum

As shown in Table 4, for chickens at 21 d, there was no effect of dietary supplementation with bilberry extract on the contents of IgA, IgY, or IgM in blood plasma (*p* > 0.05), while for birds at 63 d, supplementation with bilberry extract significantly decreased the concentrations of IgY and IgM in blood plasma (*p* < 0.05). No difference was observed in the contents of IgA, IgY, or IgM in jejunal mucosa among the treatments (*p* > 0.05).

### 3.4. Antioxidant Capacity

As shown in Table 5, for 21 d chickens, comparing with the control group, dietary supplementation with 400 mg/kg bilberry extract significantly increased (*p* < 0.05) the activity of GSH-Px in blood plasma, and GSH-Px in jejunal mucosa, where CAT activity was decreased (*p* < 0.05). Also, supplementation with 100 mg/kg bilberry extract significantly increased (*p* < 0.05) the activities of T-SOD in the jejunum, and GSH-Px in the liver, and decreased (*p* < 0.05) the MDA concentration in the liver.

For chickens at 63 d of age (Table 6), dietary supplementation with bilberry extract significantly increased the activity of GSH-Px, T-SOD, and iNOS, and decreased DAO activity and the content of MDA in blood plasma (*p* < 0.05). Compared with the controls, MDA concentration in jejunal mucosa was reduced (*p* < 0.05) by either level of dietary bilberry extract. There was no significant difference in the concentration of MDA nor activity of T-AOC in blood plasma and activities of GSH-Px, T-SOD, and T-AOC in jejunal mucosa among treatments (*p* > 0.05).

Effects of dietary supplementation with bilberry extract on antioxidant capacity of breast muscle of yellow-feathered chickens at 63 d are shown in Table 7. At 45 min postmortem, the MDA concentration in breast muscle of birds supplemented with 400 mg/kg bilberry extract was significantly lower than that of birds given 100 mg/kg (*p* < 0.05) but neither treatment differed from the controls. At 4 d post-mortem, compared with the control group, the activity of T-SOD in breast muscle was significantly increased (*p* < 0.05) by 400 mg/kg bilberry extract, and the content of MDA was significantly decreased (*p* < 0.05) by 100 mg/kg bilberry extract (*p* < 0.05). There was no significant difference in GSH-Px activity among the treatments (*p* > 0.05).

### 3.5. Meat Quality and Content of Flavor Components in Meat

As shown in Table 8, compared with the controls, pH values of breast muscle from 63 d chickens at 45 min or 4 days post-mortem were increased by dietary supplementation with bilberry extract (*p* < 0.05). Furthermore, supplementation with 100 or 400 mg/kg bilberry extract reduced the drip loss or shear stress of breast muscle, respectively. There were no significant differences (*p* > 0.05) in meat color.

Supplementation with bilberry extract in the diet did not affect the content of intramuscular fat or inosinic acid in breast muscle in chickens at 63 d (Table 9).

## 4. Discussion

### 4.1. Effects of Dietary Supplementation with Bilberry Extract on Growth Performance of Yellow-Feathered Chickens

In the current experiment, generally, dietary supplementation with bilberry extract did not affect the growth performance of yellow-feathered chickens from 1 to 63 days of age. A decreased trend (*p* = 0.067) was observed in ADFI of birds supplemented with bilberry extract, which might indicate that bilberry extract has the potential to save feed without affecting bodyweight. Kara et al. [16] reported that dietary grape procyanidins had no significant effect on ADFI and F/G of laying hens. Supplementation with different levels (0.05% and 0.1%) of thyme oil (39.9% thymol) did not affect the performance of chickens at d 28 [17]. The present findings with bilberry extract were similar, with no adverse effects on chickens, indicating the feasibility of application in chicken meat production. The cost of bilberry extract in broiler production was estimated according to the price of the material used. When supplemented with 100 mg/kg bilberry extract in diet, the average cost of feed per ton increased by USD 5, and the cost will be reduced in the future by optimizing the process of extraction.

### 4.2. Effect of Dietary Supplementation with Bilberry Extract on Immune Function of Yellow-Feathered Chickens

The thymus, spleen, and bursa of Fabricius are immune organs, the relative weights of which are important indicators of the immune function of poultry. The current study showed that dietary supplementation with bilberry extract increased the relative weight of the bursa of Fabricius of yellow-feathered chickens, indicating that bilberry extract improved the development of immune organs and likely influenced the immune function of chickens. This study was consistent with the previous results, in which Park et al. [18] found that proanthocyanidins extracted from pine bark promoted the proliferation of immune cells in the bursa of Fabricius, spleen, and thymus of chickens, reduced the expression of T cytokines and enhanced the immune function.

IgY, IgA, and IgM comprise major immunoglobulins that play a role in the neutralization of toxins, bacteria, or viruses, along with opsonization and complement activation [19]. Noh et al. [20] showed that *Platycodon grandiflorum* increased the serum levels of immunoglobulins (IgY and IgA), leading to enhanced humoral immunity. However, our results demonstrate that bilberry extract reduced the level of immunoglobulin in blood plasma. The reason for the above differences might be that bilberry extract played an antioxidant and anti-inflammatory role, thus reducing the level of oxidative stress and inflammation of chickens. Therefore, the reduced immune response led to less immunoglobulin. There is little related research, and the specific reasons and regulatory mechanisms for these results need further study.

### 4.3. Effect of Dietary Supplementation with Bilberry Extract on Antioxidant Capacity of Yellow-Feathered Chickens

The activity of DAO in blood plasma reflects conditions of damage and repair of the intestinal mucosal epithelium [21]. In the present study, bilberry extract decreased blood plasma DAO activity, consistent with the bilberry extract having a role in repairing oxidative damage. NO can be produced from L-arginine by iNOS and increased during inflammation [22,23]. Anthocyanin inhibited in vitro expression (mRNA and protein) of iNOS and also NO production in a dose-dependent manner [24]. In the present study, bilberry extract enhanced blood plasma iNOS activity that was not consistent with previous results of iNOS protein and mRNA expression, and the precise mechanisms of action should be further studied. The concentration of blood plasma NO in Hubbard layer chicks was decreased by the dietary addition of 12 mg/kg grape seed proanthocyanidin extract [25]. In agreement with the previous studies, bilberry extract decreased the concentration of blood plasma NO in chickens which suggested reduced oxidative damage because of improved antioxidant capacity.

Studies have shown that anthocyanins are absorbed into blood [26,27] and have strong antioxidant activity for chicks [25]. In the current study, dietary supplementation with bilberry extract increased activities of antioxidant enzymes including GSH-Px and T-SOD, and decreased MDA in blood and tissues in chickens so the present results were consistent with previous work. Toaldo et al. [28] found that the antioxidant capacity of humans was increased by anthocyanin-rich grape juices. Hosoda et al. [29] showed a significant increase in blood plasma SOD activity in lactating dairy cows fed anthocyanin-rich purple corn silage and Tian et al. [30] found that purple corn anthocyanins significantly improved SOD activity in the milk of goats. Placha et al. [17] demonstrated that MDA concentration in chicken blood plasma was significantly decreased by 0.1% *Thymus vulgaris* essential oil, indicating that antioxidant properties of thymol inhibited lipid peroxidation. Kara et al. [16] reported that grape proanthocyanidins improved the antioxidant capacity of laying hens and the same supplementation alleviated the oxidative stress response induced by aflatoxin B1 in chickens [31], which might reflect activating Nrf2/ARE signaling pathways [32].

The current and previous studies suggested that dietary supplementation with bilberry extract effectively reduced oxidative damage and inhibited lipid oxidation, due to the activation of antioxidant enzyme systems and enhanced antioxidant enzyme activity. Furthermore, in general, the improvement of different doses of bilberry extract on antioxidant activity of boilers was relatively effective and consistent in 63-d chickens. The reason might be that 63-d chickens were supplemented with bilberry extract for a longer time, and on the other side, organism aging and related intensification of oxidative stress might lead to a more significant role of anthocyanins.

### 4.4. Effect of Dietary Supplementation with Bilberry Extract on Meat Quality of Yellow-Feathered Chickens

PH, drip loss, and shear force are the main physical indicators for evaluating meat quality. The present study showed that the pH of breast muscle of birds supplemented with bilberry extract increased significantly. Plant extract (e.g., proanthocyanidins, carvacrol thymol) effects on meat pH are inconsistent. For example, the pH of pork was not affected by grape seed extract with proanthocyanidins as the main component [33]. The present findings of an increased pH with bilberry extract confirmed the pH of female lamb meat to have increased after dietary supplementation with oregano essential oil (83.10% carvacrol and 2.10% thymol) [34]. Additionally, bilberry extract, also decreased drip loss, possibly by inhibiting muscle glycolysis.

Shear force is an important objective indicator related to the tenderness of meat [35]. Lee et al. [36] reported that plant polyphenols (resveratrol) reduced shear force and improved the tenderness of muscle by promoting the expression of genes related to oxidative muscle fibers and inhibiting the expression of genes related to glycolytic fibers. Zhao et al. [9] found that adding 5% and 10% wine grape pomace to the diet significantly reduced the shear stress of longissimus dorsi in fattening sheep. In the present experiment, shear force was significantly decreased by supplementation with 400 mg/kg bilberry extract, indicating that an appropriate dosage of bilberry extract improved meat tenderness.

The current study showed that bilberry extract increased pH value, decreased the drip loss, and shear force of muscle, all consistent with the improved meat quality of the chickens. Combined with the positive effect of the bilberry extract on antioxidant capacity, the improved meat quality was possibly related to the increasing activity of antioxidant enzymes, which protected the structure of bio-membranes, improved the function of the antioxidant system, and maintained the integrity of muscle cells.

As an important index of chicken quality, muscle flavor reflects the contents of inosinic acid and IMF [37,38]. Previous studies have shown that muscle flavor compounds in chicken could be increased by plant extracts (flavonoids). Soybean isoflavone supplemented in diets (23 mg/kg) resulted in increasing levels of inosinic acid in thigh muscles of chickens [39]. IMF content in the breast muscle of the chickens was increased by dietary flavonoids (0.05% to 0.15% sea buckthorn fruits) [40], but there is little known of the effect of anthocyanins on muscle flavor compounds. In the present study, bilberry extract had no effect on inosinic acid and IMF in breast muscle, but further study is required.

## 5. Conclusions

Dietary supplementation with 100 or 400 mg/kg bilberry extract both generally increased activities of antioxidant enzymes in the liver, jejunal mucosa, and blood plasma, and improved several indices of meat quality despite no effect on the growth performance of yellow-feathered chickens. Receiving 400 mg/kg bilberry extract enhanced relative weight of the bursa of Fabricius when chickens were at 21 d, despite this, the effects between these two doses were generally similar. Considering the costs of production, 100 mg/kg bilberry extract was recommended when supplemented in chickens.

## Figures and Tables

**Table 1:** Composition and nutrient levels of the basal diets.

	1 to 21 d	22 to 42 d	43 to 63 d
Ingredients, %			
Corn	57.60	67.42	71.84
Soybean meal	34.20	26.00	21.20
Soybean oil	2.10	2.10	2.90
*L*-Lysine·HCl	-	0.20	0.10
*DL*-Methionine	0.15	0.18	0.16
Limestone	1.20	1.00	1.00
CaHPO_4_·2H_2_O	1.90	1.80	1.60
NaCl	0.30	0.30	0.20
Premix ^1^	1.00	1.00	1.00
Rice bran	1.55	-	-
Total	100.00	100.00	100.00
Nutrient Levels ^2^			
ME, Mcal/kg	2.90	3.00	3.10
Crude Protein, %	21.00	18.00	16.00
Lysine, %	1.16	1.06	0.85
Methionine, %	0.46	0.45	0.41
Calcium, %	1.00	0.88	0.82
Nonphytate phosphorus, %	0.44	0.41	0.37

^1^ Premix provided the following per kilogram of diets during 1 to 21 days of age: VA 15,000 IU, VD_3_ 3300 IU, VE 20 IU, VK_3_ 6 mg, VB_1_ 1.8 mg, VB_2_ 9 mg, VB_6_ 3.5 mg, VB_12_ 0.01 mg, choline chloride 500 mg, niacin 60 mg, *D*-pantothenic acid 16 mg, folic acid 0.55 mg, biotin 0.15 mg, Fe 80 mg, Mn 80 mg, Cu 8 mg, Zn 60 mg, I 0.35 mg, Se 0.3 mg; Premix provided the following per kilogram of diets during 22 to 42 days of age: VA 15,000 IU, VD_3_ 3 300 IU, VE 20 IU, VK_3_ 6.0 mg, VB_1_ 3.0 mg, VB_2_ 9.0 mg, VB_6_ 6.0 mg, VB_12_ 0.03 mg, choline chloride 1000 mg, niacin 60 mg, *D*-pantothenic acid 18 mg, folic acid 0.75 mg, biotin 0.10 mg, Fe 80 mg, Mn 80 mg, Cu 12 mg, Zn 75 mg, I 0.35 mg, Se 0.15 mg; Premix provided the following per kilogram of diets during 43 to 63 days of age: VA 10,000 IU, VD_3_ 1000 IU, VE 20 IU, VK_3_ 4 mg, VB_1_ 1.8 mg, VB_2_ 8 mg, VB_6_ 3.5 mg, VB_12_ 0.01 mg, choline chloride 500 mg, niacin 44 mg, *D*-pantothenic acid 10 mg, folic acid 0.55 mg, biotin 0.15 mg, Fe 80 mg, Mn 80 mg, Cu 8 mg, Zn 60 mg, I 0.35 mg, Se 0.15 mg; ^2^ Nutrient levels were calculated values [15].

**Table 2:** Effects of dietary supplementation with bilberry extract on growth performance of yellow-feathered chickens ^1^.

Age	Variable	CON	B100	B400	SEM	*p*-Value
	Initial BW, g	42.15	42.15	42.15	0.00	1.000
	BW, g	359.22	361.98	367.50	6.22	0.744
	ADG, g	15.16	15.21	15.48	0.30	0.747
1–21 d	ADFI, g	32.54	32.67	33.36	0.56	0.631
	F/G	2.15	2.15	2.16	0.04	0.500
	Survival rate, %	100.00	100.00	99.17	0.48	0.391
	BW, g	991.35	982.22	989.58	9.56	0.623
	ADG, g	30.10	29.46	29.55	0.33	0.173
22–42 d	ADFI, g	70.78	69.30	69.52	0.56	0.172
	F/G	2.35	2.36	2.35	0.02	0.939
	Survival rate, %	100.00	100.00	100.00	0.00	1.000
	BW g	1829.42	1815.60	1799.80	20.79	0.581
	ADG, g	39.90	39.68	38.76	0.98	0.529
43–63 d	ADFI, g	91.51	90.25	89.79	0.98	0.285
	F/G	2.30	2.27	2.32	0.06	0.595
	Survival rate, %	99.07	100.00	100.00	0.53	0.391
	ADG, g	28.76	27.85	27.74	0.37	0.387
1–63 d	ADFI, g	65.80	64.13	64.27	0.45	0.067
	F/G	2.31	2.30	2.32	0.03	0.494

^1^ Values are means of 6 replicates per treatment, each with 20 or 18 chickens. BW = bodyweight; ADG = average daily gain; ADFI = average daily feed intake; F/G = feed/gain ratio; CON = the control group; B100 = birds supplemented with 100 mg/kg bilberry extract; B400 = birds supplemented with 400 mg/kg bilberry extract; SEM = standard error.

**Table 3:** Effects of dietary supplementation with bilberry extract on the relative weight of immune organs of yellow-feathered chickens ^1^.

Relative Weight/BW, %	CON	B100	B400	SEM	*p*-Value
Liver	2.74	2.68	2.68	0.05	0.632
Spleen	0.18	0.19	0.19	0.01	0.885
Thymus	0.39	0.40	0.37	0.03	0.702
Bursa of Fabricius	0.28 ^b^	0.26 ^b^	0.32 ^a^	0.02	0.042

^1^ Values are means of 6 replicates per treatment with 2 chickens each. ^ab^ Means within a row with no common superscript differ significantly (*p* < 0.05). BW = bodyweight; CON = the control group; B100 = birds supplemented with 100 mg/kg bilberry extract; B400 = birds supplemented with 400 mg/kg bilberry extract; SEM = standard error.

**Table 4:** Effects of dietary supplementation with bilberry extract on the content of immunoglobulin of yellow-feathered chickens ^1^.

Age	Variable	CON	B100	B400	SEM	*p*-Value
	Blood plasma					
	IgA, μg/mL	6.74	6.07	6.35	0.28	0.090
21 d	IgY, μg/mL	99.97	99.56	100.00	3.59	0.996
	IgM, μg/mL	4.15	4.02	3.82	0.33	0.524
	IgA, μg/mL	6.23	6.12	6.21	0.11	0.655
63 d	IgY, μg/mL	64.11^a^	47.65 ^b^	48.51 ^b^	4.47	0.002
	IgM, μg/mL	1.97 ^a^	1.53 ^b^	1.68 ^b^	0.15	0.015
	Jejunum					
	IgA, mg/g pro	9.21	8.68	9.55	0.26	0.073
21 d	IgY, mg/g pro	67.39	68.16	64.11	2.91	0.370
	IgM, mg/g pro	8.52	8.10	8.98	0.66	0.672
	IgA, mg/g pro	1.88	2.14	2.09	0.16	0.719
63 d	IgY, mg/g pro	18.34	17.38	17.26	1.16	0.459
	IgM, mg/g pro	0.36	0.36	0.39	0.04	0.836

^1^ Values are means of 6 replicates per treatment with 2 samples each. ^ab^ Means within a row with no common superscript differ significantly (*p* < 0.05). CON = the control group; B100 = birds supplemented with 100 mg/kg bilberry extract; B400 = birds supplemented with 400 mg/kg bilberry extract; SEM = standard error; mg/g pro = mg/g protein.

**Table 5:** Effects of dietary supplementation with bilberry extract on antioxidant capacity of yellow-feathered chickens at 21 d ^1^.

Variable	CON	B100	B400	SEM	*p*-Value
Blood plasma					
GSH-PX, U/mL	1871.55 ^b^	1946.59 ^b^	2284.66 ^a^	87.24	0.031
T-SOD, U/mL	70.32	77.05	85.91	4.72	0.108
MDA, nmol/mL	1.56	1.56	1.78	0.10	0.283
Jejunum					
GSH-PX, U/mg pro	124.94 ^b^	136.12 ^ab^	143.45 ^a^	4.39	0.019
T-SOD, U/mg pro	170.98 ^b^	194.18 ^a^	181.85 ^b^	4.44	0.001
MDA, nmol/mg pro	1.23	1.38	1.30	0.08	0.511
CAT, U/mg pro	14.89 ^a^	16.20 ^a^	11.83 ^b^	0.87	0.001
Liver					
GSH-PX, U/mg pro	140.75 ^b^	157.78 ^a^	154.17 ^ab^	4.59	0.039
T-SOD, U/mg pro	419.32	399.72	392.64	7.02	0.057
MDA, nmol/mg pro	0.97 ^a^	0.63 ^b^	0.81 ^ab^	0.08	0.023
CAT, U/mg pro	55.99	53.29	50.09	2.29	0.277

^1^ Values are means of 6 replicates per treatment with 2 samples each. ^ab^ Mean values within a row with no common superscript differ significantly (*p* < 0.05). CON = the control group; B100 = birds supplemented with 100 mg/kg bilberry extract; B400 = birds supplemented with 400 mg/kg bilberry extract; SEM = standard error; GSH-Px = glutathione peroxidase; T-SOD = total superoxide dismutase; MDA = malondialdehyde; CAT = catalase; mg/g pro = mg/g protein.

**Table 6:** Effects of dietary supplementation with bilberry extract on antioxidant activity of yellow-feathered chickens at the age of 63 d ^1^.

Variable	CON	B100	B400	SEM	*p*-Value
Blood plasma					
DAO, U/L	6.85 ^a^	3.45 ^b^	3.10 ^b^	0.99	0.019
NO, μmol/L	25.88 ^a^	12.47 ^b^	29.47 ^a^	3.50	0.002
iNOS, U/mL	5.94 ^b^	8.31 ^a^	10.97 ^a^	1.13	0.012
GSH-PX, U/mL	4835.24 ^b^	6270.77 ^a^	4929.80 ^b^	288.84	0.001
T-SOD, U/mL	484.83 ^b^	856.63 ^a^	813.56 ^a^	85.66	0.006
MDA, nmol/mL	0.60	0.71	0.68	0.11	0.201
T-AOC, U/mL	5.67	4.53	5.45	0.52	0.358
Jejunum					
GSH-PX, U/mg pro	139.44	138.93	126.51	6.99	0.075
T-SOD, U/mg pro	522.25	519.72	528.11	16.37	0.951
MDA, nmol/mg pro	1.41 ^a^	0.95 ^b^	1.11 ^b^	0.16	0.032
T-AOC, U/mg pro	0.76	0.71	0.66	0.03	0.136

^1^ Values are means of 6 replicates per treatment with 2 samples each. ^ab^ Mean values within a row with no common superscript differ significantly (*p* < 0.05). CON = the control group; B100 = birds supplemented with 100 mg/kg bilberry extract; B400 = birds supplemented with 400 mg/kg bilberry extract; SEM = standard error; DAO = diamine oxidase; GSH-Px = glutathione peroxidase; T-SOD = total superoxide dismutase; MDA = malondialdehyde; T-AOC = total antioxidant capacity; mg/g pro = mg/g protein.

**Table 7:** Effects of dietary supplementation with bilberry extract on antioxidant capacity of breast muscle of yellow-feathered chickens at 63 d ^1^.

Variable	CON	B100	B400	SEM	*p*-Value
45 min post-mortem					
GSH-PX, U/mg pro	37.93	44.30	38.98	2.98	0.366
T-SOD, U/mg pro	161.12	169.66	168.24	8.12	0.602
MDA, nmol/mg pro	10.03 ^ab^	11.84 ^a^	8.57 ^b^	0.79	0.023
4 d post-mortem					
GSH-PX, U/mg pro	45.65	43.42	42.08	1.97	0.530
T-SOD, U/mg pro	143.84 ^b^	131.90 ^b^	152.38 ^a^	4.27	0.007
MDA, nmol/mg pro	88.66 ^a^	76.97 ^b^	84.44 ^ab^	7.25	0.047

^1^ Values are means of 6 replicates per treatment with 2 samples each. ^ab^ Means within a row with no common superscript differ significantly (*p* < 0.05). CON = the control group; B100 = birds supplemented with 100 mg/kg bilberry extract; B400 = birds supplemented with 400 mg/kg bilberry extract; SEM = standard error; GSH-Px = glutathione peroxidase; T-SOD = total superoxide dismutase; MDA = malondialdehyde; CAT = catalase.

**Table 8:** Effects of dietary supplementation with bilberry extract on meat quality of yellow-feathered chickens at 63 d ^1^.

Variable	CON	B100	B400	SEM	*p*-Value
L* value 45 min	51.29	51.75	52.17	0.50	0.470
a* value 45 min	11.35	11.29	10.95	0.28	0.593
b* value 45 min	11.10	10.69	10.90	0.38	0.756
L* value 24 h	49.56	49.26	49.36	0.45	0.895
a* value 24 h	11.79	11.83	11.77	0.25	0.982
b* value 24 h	10.91	9.92	10.04	0.39	0.966
pH 45 min	5.82 ^b^	5.87 ^a^	5.87 ^a^	0.03	0.024
pH 24 h	5.61	5.61	5.64	0.02	0.650
pH 4 d	5.49 ^b^	5.55 ^a^	5.57 ^a^	0.03	0.041
Drip loss, %	3.18 ^a^	2.29 ^b^	2.76 ^ab^	0.38	0.047
Shear force, N	35.81 ^a^	37.68 ^a^	31.33 ^b^	2.00	0.022

^1^ Values are means of 6 replicates per treatment with 2 samples each. ^ab^ Means within a row with no common superscript differ significantly (*p* < 0.05). CON = the control group; B100 = birds supplemented with 100 mg/kg bilberry extract; B400 = birds supplemented with 400 mg/kg bilberry extract; SEM = standard error; L* = lightness; a* = redness; b* = yellowness.

**Table 9:** Effects of dietary supplementation with bilberry extract on the content of intramuscular fat and inosinic acid in breast muscle of yellow-feathered chickens at 63 d ^1^.

Items	CON	B100	B400	SEM	*p*-Value
Intramuscular fat, mg/g	2.17	2.00	1.85	0.19	0.532
Inosinic acid, mg/g	2.61	2.26	2.22	1.94	0.127

^1^ Values are means of 6 replicates per treatment with 2 samples each. CON = the control group; B100 = birds supplemented with 100 mg/kg bilberry extract; B400 = birds supplemented with 400 mg/kg bilberry extract; SEM = standard error.

## Data Availability

The raw datasets used and analyzed during the current study are available from the corresponding author on reasonable request.
